# Reconstruction of Hand and Foot Defects with Free Serratus Carpaccio Flap and Free Serratus Fascia Flap: A Comparative Retrospective Study of Surgical Outcomes

**DOI:** 10.3390/jcm12093313

**Published:** 2023-05-06

**Authors:** Jia Wei Tee, Amir K. Bigdeli, Benjamin Thomas, Florian Falkner, Oliver Didzun, Felix H. Vollbach, Ulrich Kneser, Emre Gazyakan

**Affiliations:** Department of Hand, Plastic and Reconstructive Surgery, Burn Center, BG Trauma Center Ludwigshafen, Heidelberg University, Ludwig-Guttmann-Str. 13, 67071 Ludwigshafen, Germany

**Keywords:** free flap, microsurgery, fascial flap, foot reconstruction, hand reconstruction, serratus fascia

## Abstract

Background: Defects of the hand and foot often require an individualized reconstructive approach, due to their unique functional and aesthetic characteristics. Transferred tissues should be thin, pliable, and durable, with free fascial flaps meeting these requirements. This study aimed to evaluate the serratus fascia flap and the serratus carpaccio flap, which is a modification of the fascia flap, by including a thin muscle layer, with the goal of enhancing flap resilience and lowering morbidity rates. Methods: Between January 2000 and December 2017, 31 patients underwent microsurgical reconstruction of the hand or foot either by serratus fascia flap (fascia group) or serratus carpaccio flap (carpaccio group). The serratus fascia flaps were mainly harvested between 2000 and 2012, and the serratus carpaccio flaps between 2013 and 2017. Patient records were reviewed retrospectively, regarding demographics, complications, overall flap survival, additional revision surgeries, and surgical outcome. Categorical variables were compared using Fisher’s exact test and continuous data with the Mann–Whitney tests. Logistic regression was used to examine the correlation between comorbidities and postoperative complication rates. Differences were considered significant when *p* < 0.05. Results: The fascia group consisted of 17 patients and the carpaccio group of 14 patients. The average patient age in the fascia group was 39.2 ± 10.5 years, and it was 39.1 ± 14.7 years in the carpaccio group. Overall complication rates were significantly lower in the carpaccio group than in the fascia group (28.6% vs. 70.6%, *p* = 0.03). The rates of overall flap survival (85.7% in the carpaccio group vs. 74.5% in the fascia group, *p* = 0.66) and partial flap loss (14.3% in the carpaccio group vs. 47.1% in the fascia group, *p* = 0.07) did not differ significantly among both groups. One postoperative hematoma of the donor site requiring revision surgery was reported in the carpaccio group (7.1%, *p* = 0.45) while no donor-site morbidity was reported in the fascia group. Conclusions: Our findings indicate that the serratus carpaccio flap presents a promising alternative to the serratus fascia flap. This modification has proven to be effective in substantially reducing postoperative complications requiring additional surgical interventions. Therefore, the serratus carpaccio flap can be considered a dependable reconstructive option for addressing complex soft tissue defects of the hand and foot, with complication rates that are within an acceptable range.

## 1. Introduction

Soft tissue defects of the distal extremities pose a great challenge in reconstructive surgery. In these challenging locations, defect coverage requires an individualized reconstructive approach, due to the unique functional and aesthetic characteristics of the hand and foot [1,2]. In most cases, small-to-moderate-sized defects can be closed with locoregional flaps [3,4,5,6,7]. However, the limited mobility and availability of the adjacent skin, and, in the case of foot defects, its distinctive weight-bearing requirements, often entail a more elaborate reconstructive approach. The ideal soft tissue repair should be durable and able to provide gliding properties for the tendons in the appropriate cases. In addition, it is not uncommon that ample tissue is required to cover exposed functional structures, such as tendons, ligaments, bones, vessels, or nerves. In these situations, a free flap is often indicated. Muscle [8,9,10] but especially fasciocutaneous flaps [11,12,13] have become the mainstay for most extremity defect coverages.

Free fascial flaps display another useful alternative reconstructive option. The temporoparietal fascia flap and the serratus fascia flap provide a thin, pliable, and gliding surface [14,15]. Although they represent the thinnest available flaps, reconstructive microsurgeons are reluctant to apply them generously. Drawbacks are possible donor-site morbidity with iatrogenic alopecia after temporoparietal fascia flap harvest on the one hand, and the variations of the serratus anterior blood supply, as well as the meticulous hemostasis during serratus fascia flap harvest, on the other hand [16,17]. Another drawback is the possible failure of split-thickness skin grafts [18]. For these reasons, we have modified the harvesting technique of the serratus fascia flap by splitting the serratus muscle for easier harvest and inset since 2013 [19]. In a recent study, Rein and colleagues gave more detailed instructions on the indications and the harvesting of the modified serratus “carpaccio” flap [20]. It is hypothesized that, by including a thin muscle layer of the serratus muscle in the serratus fascia flap, damage of the fascia and vascular pedicle during flap harvesting can be minimized. In this way, the serratus carpaccio flap may be more advantageous in terms of flap survival rates and post-surgical complications.

The purpose of this study was to present our experience with the free serratus carpaccio flap in the reconstruction of soft tissue defects of the hand and foot, and to compare it with the free serratus fascia flap.

## 2. Materials and Methods

### 2.1. Study Population

All patients who underwent serratus fascia or serratus carpaccio flap surgery at the BG Trauma Center Ludwigshafen between January 2000 and December 2017 were included in this study. The study was approved by the local ethics committee of Rhineland-Palatinate (registration number: 2022-16487) and was conducted according to the STROBE guidelines (Table S1). Study inclusion criteria were: (a) soft tissue defects of the hand or foot, (b) age of patient equal to or older than 16 years, (c) indication for free serratus carpaccio flap or free serratus fascia flap, and (d) availability of complete medical records. Medical records of each patient were reviewed, with information on patient demographics, etiology of the defect, location of the defect, result of preoperative angiography, recipient vessels, microsurgical anastomosis technique, type of free flap reconstruction, and preoperative medical history. Postoperative surgical complications, including flap survival, donor-site morbidity, and medical complications were reviewed. Partial flap loss was defined as the loss of a maximum of 20% of the flap surface area (minimum of >5%).

Thirty-one cases of distal upper and lower extremity reconstructions using free serratus carpaccio or free serratus fascia flaps were identified. The patients were divided into the carpaccio group (free serratus carpaccio flap) and the fascia group (free serratus fascia flap).

### 2.2. Surgical Technique (Free Serratis Carpaccio Flap)

Patients were placed in the lateral decubitus position with the ipsilateral upper extremity freely draped. The arm was placed in a 3D support arm, to allow for unhindered movement of the arm during dissection.

**Figure 1:**
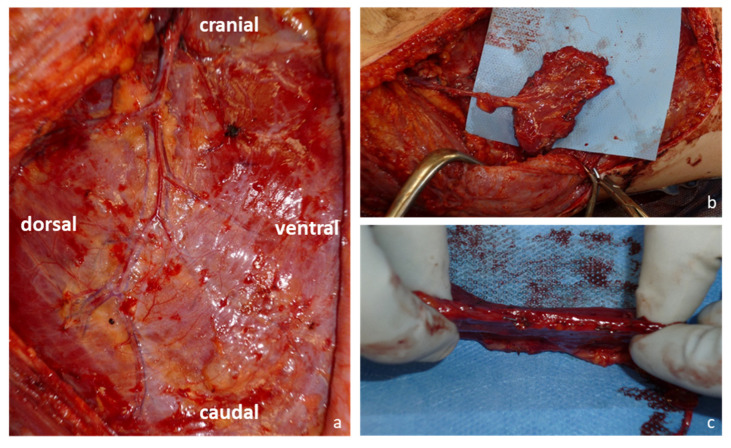
Harvesting of the serratus carpaccio flap: (**a**) Blood supply of the serratus anterior muscle and its fascia via branches from the terminal branches of the thoracodorsal artery; (**b**) Serratus carpaccio flap and its vascular pedicle after harvest; (**c**) The serratus carpaccio flap remains thin and pliable.

A vertical incision was made along the anterior border of the latissimus dorsi muscle. The latissimus dorsi muscle was retracted dorsally and dissection was performed directly to the fascia of the serratus anterior muscle. Care was taken to prevent injury of the vascular pedicle (lateral thoracic artery and serratus branches of the thoracodorsal artery) beneath the serratus fascia on the surface of the muscle (Figure 1). Then, the long thoracic nerve was identified and preserved to prevent a winging scapula. Depending on the size of the soft tissue defect, the flap was marked on the lower portion (three to four muscle slips) of the muscle. This portion was supplied by the serratus branches of the thoracodorsal artery. The serratus anterior muscle was incised between the fifth to eighth muscle slips. A muscle layer with a thickness of 3 to 5 mm was mobilized with the fascia, and dissection was continued from distal to proximal. This thin muscle layer was incorporated to prevent damage to the fascia and vascular pedicle. Flap thickness was adjusted depending on the recipient-site requirements. Smaller vascular branches within the muscle were cauterized with bipolar electrocoagulation. Only nerve fascicles running into the dissected muscle layer were cut. The vascular pedicle was dissected to an adequate length. The branches of the thoracodorsal artery to the latissimus muscle were divided to gain maximum pedicle length, if necessary. Subsequently, the flap was transferred to the recipient-site. For flap inset, we usually flipped the flap, so that the fascial area was turned over to secure the vessels and provide a better gliding surface. A split-thickness skin graft was placed on the muscle layer to cover the flap. For those patients in this study who received the serratus fascia flap, flap harvest was performed as previously described by Schwabegger and colleagues [21].

### 2.3. Statistical Analysis

Normally distributed continuous variables were presented with mean ± standard deviation (SD). Fisher’s exact test was used to examine the differences between categorical variables, while Mann–Whitney U-tests were used to compare non-normal continuous data. The presence of comorbidity variables were compared regarding incidence of postoperative complication, by calculating odds ratios with 95% confidence intervals employing logistic regression. Patients with missing data were rejected from the respective comparisons. An error probability of *p* < 0.05 was considered statistically significant. Data analysis was processed using Microsoft Excel Version 2211, R Statistical Software (v4.1.2; R Core Team 2021) and epitools R package (v0.5-10.1; Tomas J. Aragon 2020).

## 3. Results

### 3.1. Patient Characteristics

Between January 2000 and December 2017, we identified 31 patients who underwent soft tissue reconstructions at our hospital, using either serratus fascia flap or serratus carpaccio flap. Patients included 23 men and 8 women. Of these, 14 patients (45.2%) received a free serratus carpaccio flap, and 17 patients (54.8%) a free serratus fascia flap, respectively. The average patient age in the fascia group was 39.2 ± 10.5 years, whereas in the carpaccio group it was 39.1 ± 14.7 years (*p* = 0.92). The defect size ranged from 3 × 2 cm to 15 × 10 cm in the carpaccio group and from 4 × 2 cm to 15 × 15 cm in the fascia group. Without taking the missing data into consideration, the sizes of soft tissue defects in the fascia group were significantly larger as compared to the carpaccio group (96.25 ± 64.67 cm^2^ vs. 37.95 ± 35.21 cm^2^, *p* = 0.003). There was no statistical significance between the groups for a history of active tobacco use and hypertension. None of the patients had been diagnosed with pulmonary disease, cardiac disease, diabetes mellitus or peripheral vascular disease. The demographic and comorbidity variables were compared among both groups, and are shown in Table 1.

In the carpaccio group, six patients underwent surgery for traumatic injuries (42.9%), four patients for burn-related injuries (28.6%), three patients for infection (21.4%), and one patient for post-burn scar (7.1%). In the fascia group, nine patients underwent surgery for traumatic injuries (52.9%), five patients for burn-related injuries (29.4%), one patient for infection (5.9%), one patient for malignancy (5.9%), and one patient for post-burn scar (5.9%) (Table 2).

With regards to defect location in the carpaccio group, ten patients underwent flap reconstruction of the hand (71.4%), and four patients of the foot (28.6%). In the fascia group, fourteen patients underwent flap reconstruction of the hand (82.4%), and three patients of the foot (17.6%). (Table 3).

Operation time did not differ significantly between the groups (*p* = 0.13) (Table 4). The average operation time in the carpaccio group was 407 ± 112 min (range 216 to 554 min) and in the fascia group 348 ± 98 min (range 198 to 570 min). The duration of hospital stay after surgery did not differ significantly between groups (*p* = 0.07). The average duration of hospital stay was 28.6 ± 15.4 days (range 13 to 57 days) in the carpaccio group and 41.1 ± 19.2 days (range 11 to 65 days) in the fascia group.

### 3.2. Postoperative Complications

Postoperative complications of both carpaccio and fascia groups are presented in Table 2. The main finding of this study was that patients in the carpaccio group experienced significantly fewer overall complications of the flaps requiring revision surgeries compared to the fascia group (28.6% vs. 70.6%, *p* = 0.03). Total flap losses occurred in two carpaccio flaps (14.3%) due to tissue necrosis and venous thrombosis, whereas four fascia flaps (23.5%) were lost due to two events of arterial thrombosis, one tissue necrosis, and one infection (14.3% vs. 23.5%, *p* = 0.66). Partial flap loss occurred in two patients (14.3%) in the carpaccio group due to tissue necrosis, and in eight patients (47.1%) in the fascia group due to tissue necrosis (n = 7) and arterial thrombosis (n = 1) (*p* = 0.07).

One postoperative hematoma of the donor-site requiring revision surgery was reported in the carpaccio group while no donor-site morbidity was reported in the fascia group.

There was no significant correlation between the presence of comorbidity variables and the rate of postoperative complications (Table 5). The location of soft tissue defects, either in the hands or feet, did not have a significant impact on postoperative complications requiring revision surgeries in both groups (Table 3).

## 4. Discussion

Free fascial flaps were first described in the 1980s, and have been an important part of the microsurgical armamentarium in extremity reconstruction ever since [22,23]. The advantages of facial flaps include their characteristics of being thin, pliable, and well-vascularized, while providing exceptional gliding properties for underlying functional structures. The most common donor-sites are the temporoparietal and the serratus fascia [21,24,25]. Carty and colleagues evaluated the experience of a single surgeon with fascial flap reconstruction of the hand over a period of 30 years [14]. In 58% of their cases, a free temporoparietal fascia flap was used. Of all the 60 fascial flaps, an overall complication rate of 16% and no flap loss was reported. In 5% of the cases, alopecia of the donor-site was reported. A more recent study investigated the clinical outcome of 14 free temporoparietal fascia flaps for the defect coverage of fingers or the dorsum of the hand [26]. The overall complication rate was 36%, with one flap loss and one case of facial paresis. No alopecia was reported.

In our department, serratus fascia flaps were mainly performed between 2000 and 2012. This flap demonstrates similar beneficial characteristics as those of the temporoparietal fascia flap, while at the same time having a longer pedicle of up to 8 cm and a flap size of up to 20 × 15 cm [27]. Its vascular anatomy is well described [17]. In contrast, the temporoparietal fascia flap has a pedicle length of approximately 4 cm and a maximum flap size of 14 × 10 cm [28]. Overall, the serratus fascia flap is associated with low donor-site morbidity and functional deficits [29]. In a previous study, Flügel and colleagues performed free serratus fascia flaps for defect coverage of the hand in 11 cases [15]. The flap success rate was 82% and the partial flap loss rate was 9%. No secondary debulking procedures had to be performed. Ulrich and colleagues reported no flap loss in all nine cases after defect coverage of the hand. Partial flap loss was seen in 33% of the cases, which were treated conservatively. No secondary debulking procedures were needed [30].

Although the serratus fascia flap provides good functional and aesthetic outcomes, the potential drawbacks include the high partial flap loss rate and the tedious, bloody dissection. Meticulous hemostasis is crucial during the elevation of the fascia from the underlying muscle [16]. The vascular pedicle runs along the superficial surface of the fascia and eventually branches over the muscle surface. Incautious flap elevation can cause injuries to these vessels and thus lead to tissue damage and ultimately flap failure. The lack of substantial muscle tissue in the free fascial flap makes it extremely prone to blood flow obstruction, such as venous thrombosis. For this reason, the use of an ultrasonic blade was propagated to avoid damage to the flap during elevation [16].

**Figure 2:**
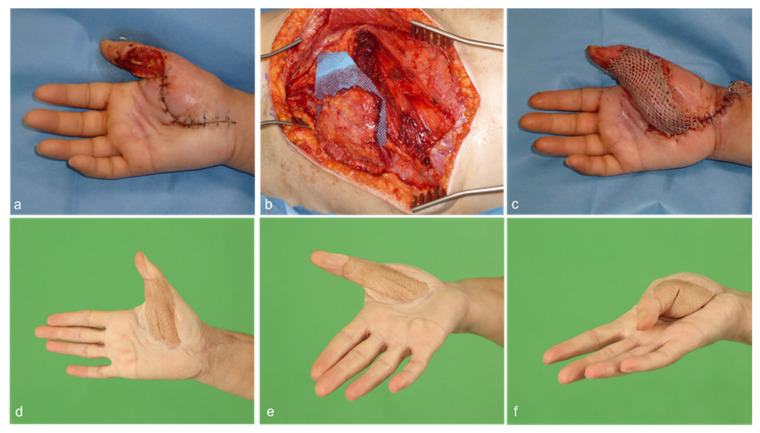
(**a**) A 51-year-old female patient with a minor knife cut of the right thumb subsequently suffered from phlegmon of the thumb and thenar region. Multiple debridement surgeries had resulted in soft tissue defect of the thumb with exposed flexor tendons as well as the 1st and 2nd digital neurovascular bundle. (**b**,**c**) A free serratus carpaccio flap raised from the right caudal portion of the serratus anterior muscle was used to reconstruct the defect. Split-thickness skin graft was used to cover the muscle. Microsurgical anastomoses were performed end-to-side to the radial artery and end-to-end to a subcutaneous vein. (**d**–**f**) After 9 months, the flap demonstrated good contour and functional result.

Taking the prevention of vascular injury into account, we modified our harvesting technique of the serratus fascia flap by retaining a thin strip of the serratus muscle. A case example is shown in Figure 2. In this way, flap elevation and thus hemostasis are performed at a safer distance, away from the serratus fascia, making the flap more durable. An adjustable thickness of muscle can be incorporated to fill up dead space at the recipient-site, if needed. Although a thin muscle layer was included in the serratus carpaccio flap, the muscle denervation eventually led to muscular atrophy, thus avoiding a bulky flap contour. Sharing the advantages of serratus fascia flaps, these harvesting techniques preserve the flap’s long pedicle of over 10 cm, with a vessel diameter between 2 and 3 mm, and its chimeric potential due to the subscapular system [19]. As with other fascial flaps, there was no need for secondary thinning procedures. This is in line with other studies for fascial flaps and the fasciocutaneous alternatives [15,26,30]. While the flap harvesting procedure varies between the free serratus fascia flap and the newer free serratus carpaccio flap, the overall surgical durations of both procedures did not differ significantly. However, it should be noted that the harvesting of the free serratus carpaccio flap has been found to be easier and involve less painstaking dissection, compared to the traditional free serratus fascia flap. In our department, there was an initial learning curve with the surgical technique for the free serratus carpaccio flap, which may have contributed to the lack of significant differences in surgical duration between the two techniques.

Success rates in both groups did not differ significantly, demonstrating reliability for these delicate areas (85.7% in carpaccio group vs. 76.5% in fascia group, *p* = 0.66). This is in line with previous studies with success rates ranging between 78.6% to 100% [15,26,30]. However, in comparison, our results show that the free serratus carpaccio flap seems more advantageous in terms of flap resistance and survival than the conventional free serratus fascia flap. The rate of partial flap loss in the carpaccio group was 14.3%, which is comparable to the typical rate seen in other workhorse free flaps [31]. Additionally, the difference between the carpaccio and fascia groups in terms of partial flap loss, although not statistically significant, suggests a trend towards a lower rate in the carpaccio group compared to the fascia group (14.3% vs. 47.1%, *p* = 0.07). We hypothesize that this is due to the modified harvest technique, which is gentle on the fascia and is less likely to injure the vessels. Other studies also reported more cases of fascial flap losses [15,30]. Overall, the need for flap revisions was significantly lower in the carpaccio group (28.6% vs. 70.6%, *p* = 0.03). Although the number of flaps was small, it seems that both flaps were more prone to complications when defects of the foot were addressed. One plausible explanation is that the lower-extremity traumatic injuries were characterized by a greater extent of tissue damage and more severe vessel trauma. Nonetheless, the carpaccio group had fewer complications, underscoring the durability of the flap. This was also confirmed in the upper extremity. Here, too, the fascia group had flap complication rates that were twice as high. Based on our results, we assume that the incorporation of a thin muscle layer protects the fascia and ensures blood circulation in the entire flap. A similar concept of vascular protection also applies to muscle-sparing free transverse rectus abdominis myocutaneous (MS-TRAM) flaps or ALT flaps, with the inclusion of a small muscle component, especially during circumstances where long intramuscular course of multiple perforators are present.

While established muscle and fasciocutaneous flaps have been used for extremity reconstructions and have demonstrated comparable results in limb salvage [32,33], recent studies have shown that fasciocutaneous flaps have fewer wound healing disorders and partial flap losses [34,35]. During the decision-making process, the reconstructive microsurgeon must consider various factors when selecting a flap, such as its ability to withstand mechanical stress and remain thin and pliable to ensure mobility. Apart from the use of free flaps, surgical developments in recent years have introduced new options for reconstructive surgery, including the use of biomaterials such as acellular dermal matrices (ADM). There have been studies exploring the potential of co-grafting ADM and split-thickness skin grafts (STSG), which could provide possible surgical options for less complex soft tissue defects [36]. Initial findings suggest that acellular dermal matrices (ADMs) hold potential for use in reconstructive surgery, and additional research is required to establish their position within current reconstructive protocols. In particular, further investigation is necessary to determine the efficacy of ADMs in the repair of soft tissue defects in the extremities [37].

Although our study demonstrates a new method of serratus fascia flap harvest with acceptable results, the limitations of the study must be critically evaluated. First, our study is limited by its retrospective monocentric design with a relatively small sample size, making it prone to observer and selection bias. The serratus fascia flap was used in our earlier period, and only in recent years has it been replaced by the serratus carpaccio flap, so patients were not randomly allocated in both groups. Further prospective studies with a larger patient cohort and a longer follow-up period are necessary to evaluate its impact as a reconstructive option on clinical and functional outcomes. Second, several reconstructive surgeons with different levels of experience over a long period of time were involved. Potential performance bias in terms of technical errors, critical indications, and strategic mistakes might be the case in one or the other free-flap complication. Third, functional outcomes of the reconstructed extremities and patient satisfaction were not assessed. Fourth, not all flap dimensions were recorded. Therefore, causal links are difficult to establish, and only speculations can be made.

In summary, we present the serratus carpaccio flap as a simpler harvesting technique aimed at increasing safety and reliability in fascial flaps.

## 5. Conclusions

Free flap reconstruction of the distal extremities remains a major challenge for the reconstructive microsurgeon who strives for excellent restoration of function and form. Among various microsurgical options, the serratus carpaccio flap can be an alternative option, offering reliability and versatility. The advantages of fascial flaps can be combined with an easier and individualized harvesting technique to address the reconstructive needs of each patient and to reduce secondary thinning.

## Figures and Tables

**Table 1:** Comparison of patient characteristics between the two groups (the carpaccio and fascia flaps). * *p* < 0.05.

	Carpaccio	Fascia	*p*-Value
No. of cases (%)	14 (45.2)	17 (54.8)	
Age, mean	39.19	39.8	0.92 (Mann–Whitney U Test)
Male:Female (% of male)	10:4 (71.4)	13:4 (76.5)	1.0 (Fisher’s Exact Test)
Hypertension (%)	1 (7)	2 (12.5)	1.0 (Fisher’s Exact Test)
Tobacco use (%)	7 (50)	2 (11.8)	0.04 * (Fisher’s Exact Test)

**Table 2:** Details of data on the microsurgical reconstruction of all the cases. M = Male, F = Female.

Patient No.	Age	Etiology	Defect Location	Defect Size	Flap Type	Flap Complications/Secondary Salvage
1	47M	Infection	Palmar hand	3.5 × 8 cm	Carpaccio	
2	37M	Trauma	Dorsum foot	3 × 3 cm	Carpaccio	
3	52F	Trauma	Palmar hand	6 × 4 cm	Carpaccio	
4	58M	Infection	Dorsum hand	9 × 3 cm	Carpaccio	
5	36M	Burn injury	Dorsum foot	6 × 6 cm	Carpaccio	
6	50F	Infection	Palmar hand	3 × 2 cm	Carpaccio	
7	58M	Burn injury	Dorsum hand	10 × 3 cm	Carpaccio	
8	17M	Trauma	Plantar foot	7.5 × 3.5 cm	Carpaccio	Total flap loss (tissue necrosis) Defect coverage with free ALT flap
9	48M	Trauma	Palmar hand	7 × 3 cm	Carpaccio	Partial flap loss (tissue necrosis) Secondary wound closure
10	39M	Trauma	Palmar hand	6 × 4 cm	Carpaccio	
11	16M	Burn scar	Palmar hand	15 × 4 cm	Carpaccio	Total flap loss (flap venous thrombosis) Defect coverage by artificial dermis (integra) and skin graft Hematoma of the donor-site requiring surgical drainage
12	27F	Trauma	Lateral foot	8 × 6 cm	Carpaccio	
13	44M	Burn injury	Dorsum hand	15 × 10 cm	Carpaccio	Partial flap loss (tissue necrosis) Defect coverage with DMCA flap
14	18F	Burn injury	Dorsum hand	6 × 7 cm	Carpaccio	
15	49M	Malignancy	Achilles region	14 × 13 cm	Fascia	Partial flap loss (tissue necrosis) Defect coverage with skin graft
16	22M	Burn injury	Achilles region	14 × 9 cm	Fascia	Total flap loss (arterial thrombosis) Defect coverage by free latissimus dorsi flap
17	42M	Trauma	Palmar hand	n. a.	Fascia	Partial flap loss (tissue necrosis) Defect coverage by skin graft
18	52M	Burn injury	Dorsum hand	n. a.	Fascia	
19	52M	Burn injury	Dorsum hand	n. a.	Fascia	Partial flap loss (tissue necrosis) Defect coverage by skin graft
20	56F	Trauma	Dorsum hand	9 × 9 cm	Fascia	Partial flap loss (arterial thrombosis) Surgical thrombectomy, vein grafting Defect coverage by skin graft
21	46M	Burn injury	Palmar hand	n. a.	Fascia	
22	42F	Trauma	Dorsum hand	7 × 6 cm	Fascia	Partial flap loss (tissue necrosis) Defect coverage by skin graft
23	25M	Burn scar	Palmar hand	n. a.	Fascia	Partial flap loss (tissue necrosis) Defect coverage by skin graft
24	26M	Trauma	Palmar hand	11 × 5 cm	Fascia	
25	35F	Trauma	Dorsum hand	12 × 10 cm	Fascia	Partial flap loss (tissue necrosis) Defect coverage by parascapular flap
26	40M	Trauma	Dorsum hand	15 × 15 cm	Fascia	Total flap loss (tissue necrosis) Defect coverage with pedicled abdominal flap
27	43F	Trauma	Palmar hand	15 × 10 cm	Fascia	Total flap loss (infection) Defect coverage by parascapular flap
28	33M	Trauma	Dorsum hand	4 × 2 cm	Fascia	
29	41M	Infection	Dorsum foot	10 × 6 cm	Fascia	Partial flap loss (tissue necrosis) Defect coverage by skin graft
30	26M	Trauma	Palmar hand	7 × 8 cm	Fascia	Total flap loss (arterial thrombosis) Finger amputation, defect coverage by local pedicled skin flap
31	46M	Burn injury	Dorsum hand	10 × 5 cm	Fascia	

ALT: anterior later thigh; DMCA, dorsal metacarpal artery.

**Table 3:** Comparison of postoperative course between the two groups (the carpaccio and fascia groups).

Group	Location of Soft Tissue Defects	Number of Flaps	Postoperative Complications Requiring Flap Revision Surgeries (%)	*p*-Value
Carpaccio	Hand	10	3 (30)	1.0 (Fisher’s Exact Test)
	Foot	4	1 (25)	
Fascia	Hand	14	9 (64.3)	0.51 (Fisher’s Exact Test)
	Foot	3	3 (100)	

**Table 4:** Risk assessment of postoperative complications requiring revision surgeries by locations of soft tissue defects. * *p* < 0.05.

Groups	Carpaccio	Fascia	*p*-Value
No. of Cases (%)	14 (45.2)	17 (70.6)	
Postoperative Complications Requiring Flap Revision Surgeries (%)	4 (28.6)	12 (70.6)	0.03 * (Fisher’s Exact Test)
Overall Flap Survival (%)	12 (85.7)	13 (76.5)	0.66 (Fisher’s Exact Test)
Flap Loss (%)	2 (14.3)	4 (23.5)	0.66 (Fisher’s Exact Test)
Partial Flap Loss (%)	2 (14.3)	8 (47.1)	0.07 (Fisher’s Exact Test)
Mean Operative Time (min)	407	348	0.13 (Mann–Whitney U Test)
Days From Surgery to Discharge	28.6	41.4	0.07 (Mann–Whitney U Test)

**Table 5:** Logistic regression analysis on the relationships of comorbidities with regard to incidence of postoperative complications requiring flap revision surgeries.

Groups			Odds Ratio	95% CI	*p*-Value
Carpaccio					
	Male:Female (% of male)	10:4 (71.4)	6.23	0.26–146.77	0.26
	Hypertension (%)	1 (7)	0.70	0.02–20.91	0.84
	Tobacco use (%)	7 (50)	0.22	0.02–2.97	0.26
Fascia					
	Male:Female (% of male)	13:4 (76.5)	0.17	0.01–3.86	0.27
	Hypertension (%)	2 (12.5)	2.62	0.11–64.70	0.56
	Tobacco use (%)	2 (11.8)	2.62	0.11–64.70	0.56

## Data Availability

The data presented in this study are available on request from the corresponding author. The data are not publicly available due to ethical, legal, and privacy issues.

## Supplementary Materials

The following supporting information can be downloaded at https://www.mdpi.com/article/10.3390/jcm12093313/s1, Table S1: STROBE Statement.

## References

[1] das De S., Sebastin S.J. (2019). Considerations in Flap Selection for Soft Tissue Defects of the Hand. Clin. Plast. Surg..

[2] Plotczyk M., Higgins C.A., Garcia-Gareta E. (2019). Skin biology. Biomaterials for Skin Repair and Regeneration.

[3] Koshima I., Moriguchi T., Etoh H., Tsuda K., Tanaka H. (1995). The radial artery perforator-based adipofascial flap for dorsal hand coverage. Ann. Plast. Surg..

[4] Angrigiani C., Grilli D., Dominikow D., Zancolli E.A. (1993). Posterior interosseous reverse forearm flap: Experience with 80 consecutive cases. Plast. Reconstr. Surg..

[5] Page R., Chang J. (2006). Reconstruction of hand soft-tissue defects: Alternatives to the radial forearm fasciocutaneous flap. J. Hand Surg..

[6] Biswas D., Wysocki R.W., Fernandez J.J., Cohen M.S. (2014). Local and regional flaps for hand coverage. J. Hand Surg..

[7] Scaglioni M.F., Rittirsch D., Giovanoli P. (2018). Reconstruction of the Heel, Middle Foot Sole, and Plantar Forefoot with the Medial Plantar Artery Perforator Flap: Clinical Experience with 28 Cases. Plast. Reconstr. Surg..

[8] Del Piñal F. (2020). An update on the management of severe crush injury to the forearm and hand. Clin. Plast. Surg..

[9] Whitney T.M., Buncke H.J., Alpert B.S., Buncke G.M., Lineaweaver W.C. (1990). The serratus anterior free-muscle flap: Experience with 100 consecutive cases. Plast. Reconstr. Surg..

[10] Kang M.J., Chung C.H., Chang Y.J., Kim K.H. (2013). Reconstruction of the lower extremity using free flaps. Arch. Plast. Surg..

[11] Adani R., Tarallo L., Marcoccio I., Cipriani R., Gelati C., Innocenti M. (2005). Hand reconstruction using the thin anterolateral thigh flap. Plast. Reconstr. Surg..

[12] Deek N.F.A.L., Hsiao J.C., Do N.T., Kao H.K., Hsu C.C., Lin C.H., Lin C.H. (2020). The medial sural artery perforator flap: Lessons learned from 200 consecutive cases. Plast. Reconstr. Surg..

[13] Narushima M., Iida T., Kaji N., Yamamoto T., Yoshimatsu H., Hara H., Kikuchi K., Araki J., Yamashita S., Koshima I. (2016). Superficial circumflex iliac artery pure skin perforator-based superthin flap for hand and finger reconstruction. J. Plast. Reconstr. Aesthetic Surg..

[14] Carty M.J., Taghinia A., Upton J. (2010). Fascial flap reconstruction of the hand: A single surgeon’s 30-year experience. Plast. Reconstr. Surg..

[15] Flügel A., Kehrer A., Heitmann C., Germann G., Sauerbier M. (2005). Coverage of soft-tissue defects of the hand with free fascial flaps. Microsurgery.

[16] Kitazawa T., Shiba M., Tsunekawa K. (2018). Free serratus anterior fascial flap combined with vascularized scapular bone for reconstruction of dorsal hand and finger defects. Case Rep. Plast. Surg. Hand Surg..

[17] Erdogmus S., Govsa F. (2005). Distal variations of the neurovascular pedicle of the serratus anterior muscle as a flap. Surg. Radiol. Anat..

[18] Buehler M.J., Pacelli L., Wilson K.M. (1999). Serratus fascia “sandwich” free-tissue transfer for complex dorsal hand and wrist avulsion injuries. J. Reconstr. Microsurg..

[19] Schoenle P., Gazyakan E., Kremer T., Harhaus L., Kneser U., Hirche C. (2018). The chimeric versatility of the subscapular system revisited: Backup options, coverage for bone transplants and vascularized lymph nodes. Plast. Reconstr. Surg. Glob. Open.

[20] Rein S., Gazyakan E., Kneser U., Kremer T. (2021). The free serratus carpaccio flap: Indications and technique. Handchir. Mikrochir. Plast. Chir..

[21] Schwabegger A.H., Hussl H., Rainer C., Anderl H., Ninkovic M.M. (1998). Clinical experience and indications of the free serratus fascia flap: A report of 21 cases. Plast. Reconstr. Surg..

[22] Wintsch K., Helaly P. (1986). Free flap of gliding tissue. J. Reconstr. Microsurg..

[23] Smith R.A. (1980). The free fascial scalp flap. Plast. Reconstr. Surg..

[24] Brent B., Upton J., Acland R.D., Shaw W.W., Finseth F.J., Rogers C., Pearl R.M., Hentz V.R. (1985). Experience with the temporoparietal fascial free flap. Plast. Reconstr. Surg..

[25] Fassio E., Laulan J., Aboumoussa J., Senyuva C., Goga D., Ballon G. (1999). Serratus anterior free fascial flap for dorsal hand coverage. Ann. Plast. Surg..

[26] Müller-Seubert W., Horch R.E., Schmidt V.F., Ludolph I., Schmitz M., Arkudas A. (2021). Retrospective analysis of free temporoparietal fascial flap for defect reconstruction of the hand and the distal upper extremity. Arch. Orthop. Trauma Surg..

[27] Zenn M.R., Jones G., Zenn M.R., Jones G. (2012). Serratus flap. Reconstructive Surgery: Anatomy, Technique, and Clinical Applications.

[28] Zenn M.R., Jones G., Zenn M.R., Jones G. (2012). Temporoparietal fascia flap. Reconstructive Surgery: Anatomy, Technique, and Clinical Applications.

[29] Fotopoulos P., Holmer P., Leicht P., Elberg J.J. (2003). Dorsal hand coverage with free serratus fascia flap. J. Reconstr. Microsurg..

[30] Ulrich D., Fuchs P., Bozkurt A., Pallua N. (2010). Free serratus anterior fascia flap for reconstruction of hand and finger defects. Arch. Orthop. Trauma Surg..

[31] Min K., Hong J.P., Suh H.P. (2022). Risk Factors for Partial Flap Loss in a Free Flap: A 12-Year Retrospective Study of Anterolateral Thigh Free Flaps in 303 Lower Extremity Cases. Plast. Reconstr. Surg..

[32] Koepple C., Kallenberger A.K., Pollmann L., Hundeshagen G., Schmidt V.J., Kneser U., Hirche C. (2019). Comparison of Fasciocutaneous and Muscle-based Free Flaps for Soft Tissue Reconstruction of the Upper Extremity. Plast. Reconstr. Surg. Glob. Open.

[33] Cho E.H., Shammas R.L., Carney M.J., Weissler J.M., Bauder A.R., Glener A.D., Kovach S.J., Hollenbeck S.T., Levin L.S. (2018). Muscle versus Fasciocutaneous Free Flaps in Lower Extremity Traumatic Reconstruction: A Multicenter Outcomes Analysis. Plast. Reconstr. Surg..

[34] Lee Z.H., Abdou S.A., Daar D.A., Anzai L., Stranix J.T., Thanik V., Levine J.P., Saadeh P.B. (2019). Comparing Outcomes for Fasciocutaneous versus Muscle Flaps in Foot and Ankle Free Flap Reconstruction. J. Reconstr. Microsurg..

[35] Thomas B., Warszawski J., Falkner F., Nagel S.S., Vollbach F., Gazyakan E., Schmidt V.J., Kneser U., Bigdeli A.K. (2021). A Retrospective Comparative Functional and Aesthetic Outcome Study of Muscle versus Cutaneous Free Flaps for Distal Upper Extremity Reconstruction. J. Reconstr. Microsurg..

[36] Petrie K., Cox C.T., Becker B.C., MacKay B.J. (2022). Clinical applications of acellular dermal matrices: A review. Scars Burn. Heal..

[37] Gierek M., Łabuś W., Kitala D., Lorek A., Ochała-Gierek G., Zagórska K.M., Waniczek D., Szyluk K., Niemiec P. (2022). Human Acellular Dermal Matrix in Reconstructive Surgery—A Review. Biomedicines.

